# Supplementary material for: Trialling the efficacy of a technological visuo-cognitive training program as a compensatory tool for visual rehabilitation after stroke: A pilot study
Source: PLOS Digit Health. 2026 Feb 23;5(2):e0000781. doi: 10.1371/journal.pdig.0000781 (PMC12928402; doi:10.1371/journal.pdig.0000781)
Supplement: Attachment — Submitted filename: Reviewer Comments_SD (4).docx [file pdig.0000781.s001.docx]

**Response to Reviewers**

We are grateful for the thoughtful and constructive feedback provided by the editor and both reviewers. Your comments prompted a comprehensive re-evaluation of our analyses and presentation, which we feel has substantially strengthened the manuscript. In response to the depth of methodological refinement requested, we have re-run both our quantitative and qualitative analyses, implementing multiple coding procedures for the qualitative data to ensure reliability and robust reporting standards throughout. We thank the reviewers for their patience in permitting us to conduct these analyses. Below, we provide detailed responses to each comment, along with a summary of the revisions made to the manuscript. Line numbers cited refer to the clean version of the manuscript.

-------------------------------------------------------------------------------------------------------------

**Editor Comments**

1.) Not clear why acceptability and feasibility would or could be assessed before patients interact with the intervention – please clarify.

We thank the reviewers for bringing this to our attention. The Results section has been overhauled to reflect this (L366) and now makes clear that these constructs were reviewed after the intervention took place (L376).

2.) Similarly, it’s not clear how the System Usability Scale could have been collected at baseline, given that participants would not have had experience with the intervention yet. So, they would be answering items like “I thought the system was easy to use” without having actually used the system? Please check.
We recognise that this was confusing and the oversight has been corrected. Now, only post-intervention descriptives are reported (L376).

3.) Clarity would be improved if the design subsection of the methods section more directly and clearly stated that this study is a single-arm pre-test, post-test design

We have revised the Design subsection to explicitly state that this is a single-arm pre-test, post-test design study (L144).

4.) The fact that the follow-up assessment was not collected at a specific timepoint - authors should note this, as well as report descriptive statistics (including a range) of the time since baseline that follow-ups were collected
We thank the reviewer for highlighting this point. To clarify, all participants completed their follow-up assessment at a fixed timepoint: baseline assessment on Day 0, five training sessions on alternate days (Days 1–11), and a final follow-up assessment on Day 12. There was therefore no variation in the timing of follow-up assessments. We have revised this to make this clearer in the manuscript in the Clinical Assessments section (L167)

5.) I do not believe the Intrinsic Motivation Inventory assesses either usability or accessibility. What was this measure used to assess, then? In addition, what measures or approaches were used to assess feasibility and acceptability? Please clarify

We recognise that the way in which this was framed was confusing, and as part of the Results overhaul, we explicitly discuss Feasibility, Acceptability, and Usability in terms of the measures used to calculate them (if any).

We are clear that Feasibility lacks an objective measure other than completion of sessions and adherence to requirements thereof. We are also now explicit that the SUS was used to measure usability (L215), and we report the mean score with SD and range, relating this to the standards set out by Bangor et al. (2009) (L376)

For Acceptability, we detail in the methods (L232) and reiterate under the aforementioned subheading that the IMI, although not a bespoke acceptability measure, conceptually overlaps with the constructs of interest when assessing acceptability (L367).

6.) Authors should state what approach they used to qualitative analyses and include a reference. Was any other author involved in coding other than AF? Was agreement upon codes assessed? I believe most well-accepted approaches to qualitative analysis call for >1 coder.

Due to both reviewers highlighting concerns regarding the qualitative data analysis, we have re-coded this data when revising our manuscript and clarified our qualitative analytic approach in the Methods section (L271). We specify that we used Template Analysis following Brooks et al., (2015) guidelines, and we have added the appropriate references. We also clarify that the analysis was led by SD with involvement from AF. Both authors independently coded all ten transcripts using an agreed deductive–inductive framework, with flexibility to add emergent codes. Although Template Analysis does not rely on formal inter-rater reliability, we ensured analytic rigour through regular consensus meetings to compare interpretations, discuss discrepancies, and refine code definitions.

7.) Whenever the means of two scores are compared, they should clearly identify which mean is pre-test and which is post-test, and include standard deviations for each. This applies to Table 1 & Table 2, as well.

We have thoroughly reviewed and revised the quantitative results presentation to address this concern. Specifically, we have clearly labelled our tables with pre-assessment and post-assessment columns, with standard deviations provided for each measure at both timepoints. All means are now presented in the format M (SD) for both pre- and post-assessment conditions (see Table 2 and Table 3). We have also revised the text describing all quantitative results to explicitly identify which mean corresponds to pre-test and which to post-test when comparisons are made. For example, comparisons now clearly state means and standard deviations for pre and post assessment for each measure reported (e.g. L312).

8.) In the first paragraph of the Results section, the authors report that “there were no further significant differences found for other items.” The authors should specifically report each item they evaluated – focusing on only “significant” differences can make it seem as though the collected evidence is stronger, just by weight of emphasis.

We have changed this so that each result is explicitly reported, significant or not (e.g. L315).

9.) In the discussion, the authors conclude that the system was “usable,” which may be due to post-test SUS scores alone – but these raw scores do not appear to be reported. Please make sure these scores are reported.
We are also now explicit that the SUS was used to measure usability, and we report the mean score with SD and range, relating this to the standards set out by Bangor et al. (2009) (L376).

10.) The basis on which the authors conclude that the intervention was “accessible” is not entirely clear –this is a specific term with specific meaning. Please clarify.

We have removed any information about accessibility and reframed according to acceptability and usability. Accessibility itself is inferable through feasibility, which acknowledges that, despite mobility issues presenting a minor barrier, engagement with the system as a whole was possible in all cases (L532).

 -------------------------------------------------------------------------------------------------------------

**Reviewer 1 Comments**
*General comments*
The Discussion would benefit from expanded discussion of several factors, including the time post-stroke of the participants (reducing the potential confound of spontaneous recovery), as well as the validation of the Sensory Station in other disease models.

We have substantially expanded the Discussion to address these concerns. Regarding time post-stroke, we have now explicitly discussed our cohort's heterogeneous time-since-stroke distribution (1-30 years, M ≈ 11 years) and how this reduces the potential confound of spontaneous recovery. With many participants several years post-stroke, natural recovery processes are unlikely to explain the observed improvements. The revised Discussion explains how time since stroke moderated participant expectations, with those many years post-stroke expressing realistic expectations while recognising potential value (L549). We have also expanded discussion of evidence showing that visual impairments are often missed early in stroke recovery and argue that earlier intervention delivery may facilitate greater neuroplasticity-dependent recovery (L553). This positions timing of intervention delivery as a critical variable warranting systematic investigation in future research.

Regarding validation of the Sensory Station, the revised Discussion references the application and validation of technological visuo-cognitive training in other neurological populations (including Parkinson's disease), providing important context for the validity and feasibility of technology-based visual rehabilitation more broadly (L567). This situates the SSS within an established framework of validated rehabilitation tools with evidence across multiple disease contexts, while acknowledging that further stroke-specific validation is warranted.

There is now substantial (if controversial) literature regarding visual rehabilitation training following stroke to improved quality of life and reduce the visual deficit. It is unclear where the SSS and TVT in general fit into this growing field.

We acknowledge the reviewer’s comment regarding the broader literature on visual rehabilitation. In the revised introduction, we now more clearly situate the SSS and TVT within the ongoing debate on restitution, substitution, and compensation approaches. We highlight that our study examines a novel tool with compensatory and visuo-cognitive elements and thus contributes to the growing field of digital rehabilitation for post-stroke visual loss (L106).

Is there any proposed mechanism or theory behind the benefit of this training? While this is a limited pilot study, there is limited motivation beyond clinical benefit, which makes it unclear the real benefit of the intervention. Would stroke patients without visual impairments receive a similar benefit simply from performing cognitively demanding tasks and learning to interact with the technology? A unifying theory of why this training is expected to be beneficial would aid in interpreting the outcomes.

We have added discussion of potential mechanisms by which TVT may benefit stroke survivors with visual field loss, including enhancement of compensatory scanning, attentional control, and visuo-cognitive skills through repeated practice. While the present pilot study is not designed to directly test these mechanisms, clarifying this rationale strengthens the theoretical basis for our investigation (L109)

*Specific comments*
SSS should be defined in the abstract.
The SSS has been defined in the abstract, as well as in the first instance in the main body (L124).

The “Participants” heading under Results is uninformative and confusing, given the “Participants” heading listed again under Methods.
The “Participants” subheading under Results has now been removed, and the Results section has been restructured for improved clarity and to avoid redundancy.

Formatting of Table 3 and Table 4 is causing some odd line overruns, making them a little hard to read.
We have reformatted these tables to try to avoid this issue

It is unclear how “Impairment” is defined in Table 3 for each of the listed categories.

To improve clarity, we have amended the table caption and text to explicitly state that “Impairment” refers to participants’ self-reported stroke-related difficulties, gathered during the demographic and visual health assessment. This has been clarified in the Methods section (L175) and Table 1 caption (L164).

-------------------------------------------------------------------------------------------------------------

**Reviewer 2 Comments**
*Major Comments*
1. Interpretation of key outcome measures (Fatigue & Falls Concern)
The paper reports on improvements in fatigue and fear of falling; however, as currently presented, the FES-I and FSS scores appear to increase post-intervention, which (according to standard scoring conventions) would indicate worsened outcomes. This may reflect a mislabelling or misordering of scores, but it is difficult to determine without “pre” and “post” labels. The directionality and scoring of these scales should be explicitly described and correctly interpreted in both the Results and Discussion sections.

After recompleting the analyses and ensuring variables were computed and entered correctly, a significant decrease in fatigue is observed, alongside a nonsignificant downward trend for fear of falling. Directionality is now explicitly mentioned in both cases (L321).

2. Overstating efficacy in an uncontrolled, underpowered design
The manuscript uses language suggestive of clinical improvement or causal inference (e.g., “improved functioning,” “demonstrated efficacy”), which is not supported by the study’s design. The absence of a control group, the small and heterogeneous sample (n = 10; stroke onset 1–30 years), and the exploratory nature of the study limit the strength of any claims. More tentative phrasing (e.g., “observed changes” or “preliminary findings”) would be more appropriate throughout. The limitations section does briefly note that multiple comparisons were made, but it does not indicate whether any correction was applied or considered. Given the number of outcomes tested, this increases the risk of Type I error. If no correction was applied, the rationale should be clearly stated (e.g., due to the exploratory nature of the study). Additionally, the abstract highlights statistically significant findings using p < .05 values, without clarifying that these are uncorrected. This may overstate the robustness of the results. If uncorrected thresholds are reported, this should be clearly acknowledged.

We recognise that the terminology used could have been misleading. The mentioned sections have been overhauled to reflect the necessarily tentative nature of any claims made. Furthermore, the decision has been made to abstain from conducting corrections: due to the pilot quality of the study, we find direction and magnitude of observed effects to be more useful than employing conservatism in interpreting significance. Indeed, a study of this design is very unlikely to have the statistical power to detect significant effects. We have explicitly acknowledged these uncorrected thresholds in the statistical analysis section of the methods section (L260).

3. Transparency and rigor in qualitative analysis
Thematic analysis is a central component of this mixed-methods study. However, it appears that coding was conducted by a single researcher, with no mention of inter-coder reliability, peer debriefing, or reflexivity. While adding a second coder retrospectively may not be feasible, the authors should (1) explicitly acknowledge this limitation, (2) add a brief reflexivity statement regarding the coder’s background and potential biases, and (3) describe any procedures used to support the trustworthiness of the analysis (e.g., consultation with co-authors or iterative team discussions). These steps would strengthen confidence in the qualitative findings.

Due to both reviewers highlighting concerns regarding the qualitative data analysis, we have re-coded this data when revising our manuscript. We have revised the manuscript to clarify our analytic procedures and strengthen the transparency of the qualitative component. In response, we have (1) added a brief positionality statement (L282), (2) clarified that the dataset was re-analysed using a two-coder approach (L271), and (3) described the steps taken to support trustworthiness (L278).

Specifically, although author 1 coded the initial dataset, due to resource constraints the revised analysis involved author 2 as the primary coder with author 1 providing secondary coding input. Both authors independently coded all transcripts over this revision period and met regularly to compare interpretations, resolve discrepancies, and refine code definitions. Consistent with Template Analysis, we did not calculate inter-coder reliability; instead, we prioritised collaborative reflexive dialogue to enhance rigour. We have also added a concise positionality statement acknowledging authors background and potential influences on interpretation as coders.

4. Limited engagement with relevant literature on digital interventions for hemianopia:

While the authors reference one prior study involving a digital tool for visual rehabilitation, the discussion of related work remains narrow. Key studies such as those related to the Eye-Search project (e.g., Leff et al., 2020) are not included, nor are feasibility studies involving gamified interventions for children with hemianopia (e.g., Ivanov et al., 2018; Waddington et al., 2018). Although these studies primarily focus on oculomotor training (unlike the cognitive training approach used in this study) they establish important precedents for the feasibility, engagement, and translational potential of digital tools in visual rehabilitation. Broadening the literature review and discussion to reflect this wider body of work would help position the current intervention more accurately within the landscape of digital rehabilitation research for hemianopia.

We have broadened our introduction to incorporate discussion of related work, including the Eye-Search project (Szalados et al., 2021) and feasibility studies of gamified interventions for children with hemianopia (Ivanov et al., 2018; Waddington et al., 2018). These examples demonstrate that structured and gamified training approaches are feasible and acceptable across populations, can enhance engagement with rehabilitation, and may improve both objective visual outcomes and patient-reported experiences. We believe this addition strengthens the rationale for our focus on evaluating the usability and acceptability of a digital visuo-cognitive training intervention for stroke survivors with visual loss. (L112)

*Minor comments*
Senaptec System Validity: The manuscript does not address the psychometric properties of the Senaptec Sensory Station tasks, which form the basis of the intervention and outcome measurement. While this is acceptable in a pilot context, the authors should clarify whether these tasks are validated neuropsychological measures or proprietary tools without established reliability or construct validity. A brief acknowledgment one way or the other would enhance transparency.
The evidence regarding the psychometric properties of the Senaptec Sensory Station is currently limited. We have now clarified this in the Design section of the manuscript. Specifically, we note that while one study in healthy, college-aged individuals reported good reliability for tasks such as go/no-go, multiple-object tracking, eye–hand coordination, depth perception, and reaction time, other visual skill assessments demonstrated poor reliability (Zynda et al., 2024), and these findings have not yet been examined in clinical populations. As the reviewer highlights, this limitation is acceptable within the context of a pilot study, and we have made clear that our primary aim was to explore usability and acceptability, with psychometric validation identified as an important avenue for future research (L151).

Cognitive Inclusion Criteria: The paper states participants had "good cognitive ability" but does not clarify how this was determined. Please clarify whether a formal screening tool or clinical judgment was used.

We did not use a formal cognitive screening tool in this pilot study. Instead, cognitive ability was assessed using clinical judgment to ensure participants could follow instructions and provide informed consent. To clarify, we have amended this in the Participants section to read: *“All participants had been diagnosed with a non-progressive left or right visual field defect, demonstrated sufficient cognitive ability as determined by clinical judgment, and were able to provide informed consent.”* (L158)

Terminology Clarification: The use of the term “pseudo-random” to describe task ordering is unclear. Please indicate whether this refers to a fixed sequence, partial counterbalancing, or some other approach.

To clarify, the task order was counterbalanced across participants to minimise potential order effects. As this was a small pilot study, full counterbalancing was not feasible, but the order was varied systematically so that no participant completed tasks in the same fixed sequence. We have updated the Methods section to read: *“Assessment tasks were counterbalanced across participants to minimise potential order effects.”* (L178)

Abstract and Introduction: Some sentences are verbose or repetitive. For example, “post-stroke visual field loss is a common impairment after stroke” could be condensed. Consider revising for conciseness and clarity.

We have revised both the Abstract and the Introduction to improve clarity and conciseness, removing redundant phrasing and condensing overly long sentences. For example, the sentence highlighted by the reviewer has been amended to a more concise form, and similar changes have been made throughout these sections.

Motivational Measures: The Intrinsic Motivation Inventory is an appropriate tool, but the version used and any adaptations made for this population should be described.

We have revised the Methods section to include detailed description of the specific version of the Intrinsic Motivation Inventory used in this study (L232).

Table Formatting: Statistical tables should be checked for completeness and alignment with journal expectations. In particular, they should include degrees of freedom, and ideally effect sizes and confidence intervals, to support interpretability.

We have revised all statistical tables to align with the journal’s formatting expectations and have added degrees of freedom, along with effect sizes and confidence intervals to improve clarity and interpretability
